# Assessing the impacts of vaccination and viral evolution in contact networks

**DOI:** 10.1038/s41598-024-66070-5

**Published:** 2024-07-08

**Authors:** Rodolfo Blanco-Rodríguez, Josephine N. A. Tetteh, Esteban Hernández-Vargas

**Affiliations:** 1 Department of Mathematics and Statistical Science, University of Idaho, Moscow, ID 83844-1103 USA; 2 Institute for Modeling Collaboration and Innovation, University of Idaho, Moscow, ID 83844-1103 USA; 3 Frankfurt Institute for Advanced Studies, 60438 Frankfurt am Main, Germany

**Keywords:** Applied mathematics, Computational models

## Abstract

A key lesson learned with COVID-19 is that public health measures were very different from country to country. In this study, we provide an analysis of epidemic dynamics using three well-known stochastic network models—small-world networks (Watts–Strogatz), random networks (Erdös–Rényi), and scale-free networks (Barabási–Albert)—to assess the impact of different viral strains, lockdown strategies, and vaccination campaigns. We highlight the significant role of highly connected nodes in the spread of infections, particularly within Barabási–Albert networks. These networks experienced earlier and higher peaks in infection rates, but ultimately had the lowest total number of infections, indicating their rapid transmission dynamics. We also found that intermittent lockdown strategies, particularly those with 7-day intervals, effectively reduce the total number of infections, serving as viable alternatives to prolonged continuous lockdowns. When simulating vaccination campaigns, we observed a bimodal distribution leading to two distinct outcomes: pandemic contraction and pandemic expansion. For WS and ER networks, rapid mass vaccination campaigns significantly reduced infection rates compared to slower campaigns; however, for BA networks, differences between vaccination strategies were minimal. To account for the evolution of a virus into a more transmissible strain, we modeled vaccination scenarios that varied vaccine efficacy against the wild-type virus and noted a decline in this efficacy over time against a second variant. Our results showed that vaccination coverage above 40% significantly flattened infection peaks for the wild-type virus, while at least 80% coverage was required to similarly reduce peaks for variant 2. Furthermore, the effect of vaccine efficacy on reducing the peak of variant 2 infection was minimal. Although vaccination strategies targeting hub nodes in scale-free networks did not substantially reduce the total number of infections, they were effective in increasing the probability of preventing pandemic outbreaks. These findings underscore the need to consider the network structure for effective pandemic control.

## Introduction

In our globally interconnected world, pandemics are both an inevitable and unpredictable challenge. This inherent unpredictability complicates our efforts to combat emerging pandemics effectively. However, the lessons learned from past pandemics can better prepare to confront future outbreaks. Morse et al.^1^ have thoroughly examined the intricacies of pandemic control and prediction while highlighting the critical nature of preemptive strategies. In particular, the COVID-19 pandemic served as a stark reminder of our unpreparedness for emerging pandemics and underscored the urgency of preparing the scientific community for the next outbreak^2^. Recent studies have harnessed the power of machine learning to predict impending pandemics, explored innovative drug research, and offered hope for the prevention of widespread infections and associated fatalities. However, given the apparent impossibility of predicting emerging pandemics, we are left with the question of what preventive strategies are adequate to mitigate the spread of a disease within a population.

A person infected with a respiratory disease can spread the virus by exhaling, talking, coughing, and sneezing creating the potential for transmission over longer distances. This was recently exposed with the rapid spread of COVID-19 throughout the world, hence great efforts have been made to know the forms of transmission concluding that the main one is airborne transmission^3^. Therefore, non-pharmaceutical interventions such as hand washing and social distancing are needed in addition to preventive measures such as effective ventilation, air filtration, and the use of appropriate face masks^4^. Given these concerns surrounding possible future pandemics, computational studies related to epidemiological phenomena have been a simple tool to model the behavior of diseases under certain conditions, with SIR (Susceptible-Infected-Removed) models being the preferred ones^5^. Models based on differential equations are widely used in epidemiological studies^6^. Although these deterministic models give great results, they cover the fact that the process of pathogen transmission has stochastic ties, and that it is dominated by a few highly connected individuals in a population^7^. Consequently, studies of the propagation of a disease through a network dictated by its connections (edges) are widely used^8^. Network-based epidemiological models use graph theory to represent interactions between individuals, where nodes represent individuals, and edges symbolize connections or contacts between them; the state of the agents can be modeled with a SIR model, for example. Three common network models utilized in epidemiological studies are the Watts–Strogatz (WS) network^9^, Erdos–Renyi (ER) network^10^, and Barabási–Albert (BA) network^11^. The WS network involves nodes connected to their nearest neighbors with some randomly rewired edges that make it suitable for simulating communities with localized connections. On the other hand, the ER network is fully random with each pair of nodes having a fixed probability of being connected that makes it useful for studying more homogenously connected populations. The BA network follows a scale-free structure where a few highly connected nodes (hubs) coexist with many less connected nodes that resemble real-world scenarios like social networks and transportation systems^12,13^. It is important to note that the structure of the network should be considered since it dictates the dynamic of the disease within the population^14^.

**Table 1:** Vaccine efficacy of Pfizer-BioNTech vaccine for the omicron variant of SARS-CoV-2 and corresponding $\kappa$-ratios

Week after vaccination	Vaccine efficacy (%)	$\kappa$-ratio
2–4	65.5	0.690
5–9	48.7	0.513
10–14	30.1	0.317
15–19	15.4	0.162
20–24	11.5	0.121
$\ge$ 25	8.8	0.093

Adapted from Andrews et al.^44^.

**Table 2:** Total number of infections throughout the entire pandemic during the lockdown scenarios, averaged from 100 replicates, for the three networks and categorized by variant infection.

	Total number of infected nodes ($10^5$)					
	WS		ER		BA	
	*variant 1*	*variant 2*	*variant 1*	*variant 2*	*variant 1*	*variant 2*
No lockdown:						
	1.77	1.73	1.63	1.69	1.21	1.30
Continues lockdown:						
*30 days*	1.52	1.85	1.52	1.70	0.92	1.22
*90 days*	1.58	1.56	1.44	1.70	0.90	1.17
*150 days*	1.54	1.49	1.22	1.51	1.00	1.29
Lockdown by intervals:						
*7 days*	1.53	1.67	1.41	1.58	1.00	1.27
*15 days*	1.56	1.52	1.46	1.47	0.92	1.19
*30 days*	1.54	1.35	1.44	1.38	0.88	1.30

With respect to social distancing, the question arises as to how strict this should be and for how long it should be maintained in order to maintain a minimum level of stress in the population. During the COVID-19 outbreak, some public health measures were studied and various strategies were proposed to help mitigate the spread of SARS-CoV-2 that were considered realistic achievable scenarios. Utilizing SIR-type models, Sereno et al.^15^ explored strategies to minimize the total number of infections while simultaneously managing the peak prevalence of infected individuals and mitigating the impact of non-pharmaceutical interventions. Afshar-Nadjafi et al.^16^ simulated different stochastic networks to study seesaw lockdown scenarios with different levels of constraint, and they showed that the outbreak can be flattened by up to 120%, i.e., the duration of the peak can be shifted from 42 days to 92 days under softer alternatives instead of a complete restriction. Syga et al.^17^ assume a small-world network that allows distinguishing local contacts and random contacts in the population and show that the non-pharmaceutical interventions to mitigate the COVID-19 outbreak reduced random contacts in the transmission network, and it resulted in a transition from an exponential to a constant regime of new cases.

On the other hand, when a disease becomes pandemic, the need arises to immunize the population through the development of vaccines. For COVID-19, at least four companies produced vaccines approved for emergency use either by the US Food and Drug Administration or by the European Medicines Agency: Pfizer-BioNTech, Moderna, AstraZeneca, and Johnson & Johnson/Janssen Pharmaceuticals^18^. It has become clear that a sustainable way of preventing widespread morbidity and mortality relies on a combination of mass vaccinations and non-pharmaceutical interventions such as lockdowns and social distancing^19–23^. However, in the absence of vaccination and with relaxed social distancing measures, the SARS-CoV-2 virus demonstrated the ability to mutate and develop variants^24,25^. Some of these variants had multiple genetic changes which led to phenotypic changes increasing transmissibility and mortality and had the potential to reduce the effectiveness of vaccines^26^. The mass deployment of highly effective vaccines, while the infection is widespread, may rapidly exert selection pressure on the SARS-CoV-2 virus directed towards mutations that escape the vaccine-induced immune response^24,27^. However, the strength of this selection and the likelihood of vaccine escape is unknown at this time. Thus, some of these vaccines (Pfizer-BioNTech and Modern) have shown high efficacy for the original strain of the virus, the emergence of SARS-CoV-2 variants of concern has cast a shadow over the expectation of a swift end to this pandemic. Variants of concern, especially delta, alpha, gamma, and omicron, are the main reason for continued infections globally^28,29^. Consequently, even though mass vaccination campaigns have been launched in many countries including Israel, Germany, the United Kingdom, and the United States with more than 50% of the population fully vaccinated^30^, the continued evolution of SARS-CoV-2 could eventually give rise to a fully vaccine-resistant variant^31^. Such a variant has the potential to spread quickly due to its ability to infect vaccinated, recovered, and fully susceptible individuals consequently reducing the efficacy of current SARS-CoV-2 vaccines^26,32^.

Despite numerous prior investigations into virus transmission and various vaccination strategies to mitigate pandemics, our understanding of how network structure influences disease spread dynamics remains incomplete. In the challenging task of inferring precise network structures within populations, complexities abound^33^. While research indicates that real-world social and encounter networks in regions, like Great Britain^34,35^, can be accurately modeled using scale-free networks. It is crucial to acknowledge that actual complex networks are dynamic and can undergo structural changes over time. Consequently, the effectiveness of these strategies in diverse network types, such as varying societal contexts, remains an open question. In previous work, Tetteh et al.^36^ used two different models of stochastic networks where they were able to recapitulate Italian SARS-CoV-2 data from February 2020 to September 2020. In addition, they studied two different vaccination strategies with different percentages of vaccinated population and concluded that at least 70% of a given population should be vaccinated to obtain herd immunity, independently of the network structure^36^. On the other hand, scale-free networks exhibit a distinctive feature of highly connected nodes leading researchers to propose strategies such as lockdowns and targeted immunization focused on these hubs^14,37,38^. However, it is worth noting that these studies often consider networks either a small number of nodes, or a low degree of node connectivity, or both. Moreover, in some cases, the connections of hub nodes are partially or completely eliminated during the simulation of mitigation strategies. In real-world networks, connections between nodes are not entirely eliminated even if a node is immunized; there remains a possibility of an immunized node getting infected and subsequently infecting other nodes, albeit with reduced probability.

In this paper, we studied the infectivity of a virus in a population model with the three most popular stochastic network models used in network-based epidemiological studies, and we considered the introduction of a more vaccine-resistant variant in circulation with the original wild-type virus. Different lockdown and vaccination strategies were developed to evaluate the impact of the network structure with the dynamic of the disease.

## Materials and methods

### Network models

In this work, we simulated the transmission of a virus with two variants. We used complex networks with *N* nodes in different states of the disease. The networks considered are listed below:

- *Watts–Strogatz* (WS) network^9^: This network can be highly clustered and models local contacts among the nearest neighbors and random contacts among distant neighbors considering a rewired probability;
- *Erdös–Rényi* (ER) network^10^: This network is completely random where each node is equiprobably linked to another node. It is equivalent to the WS network when the latter has a rewired probability of 1;
- *Barabási–Albert* (BA) network^11^: It is a random scale-free network and generates nodes with a high number of contacts (degree) since this network weights the probability of connection according to the degree of the nodes.

**Figure 1:**
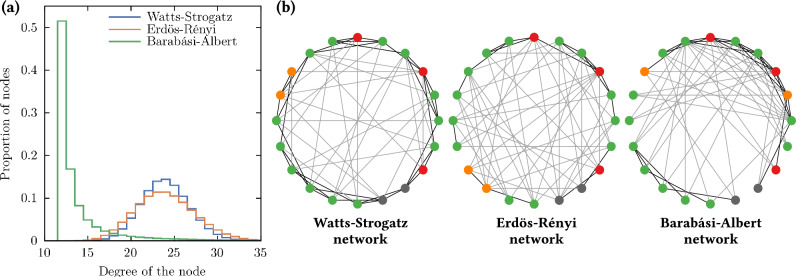
On the left side, (**a**) the graph shows the distribution of the node degree for the three different networks used in this work. The average degree is 24 and is the same for all three networks. On the right side, (**b**) the representation of the three different networks using 20 nodes and an average degree of 6: the Watts–Strogatz (WS) network with $p = 0.4$; the Erdös–Rényi (ER) network; and the Barabási–Albert (BA) network. The black lines represent the local contact with the nearest neighbors and the grey lines represent a random contact or distant contact. We can observe the local clusters in the WS network, the lack of local contacts in the ER network, and the high degree of some nodes in the BA network. The color of the nodes represents the state that they are in: exposed (green), asymptomatic (orange), infected (red), and removed (gray).

The three different networks used are heterogeneous where their edges (contacts between nodes) are generated randomly at the beginning of the simulations. Figure 1a shows the distribution of the node degree for the three different network models with the same average degree; the WS network and ER network have a normal distribution whereas the BA network is a power law distribution. In all our simulations the number of nodes *N* was fixed to $10^6$, this number approximates the population of a typical large city. We tested different numbers of nodes, ranging from $10^4$ to $10^8$, and noticed variations primarily on the day of the infection peak. In ER-type networks, the smaller the population, the earlier the peak. We considered that using one million nodes provides a practical and informative basis for understanding larger populations in a reasonable computational timeframe.

To generate WS and ER networks, we set up *N* nodes in a one-dimensional loop (arranged in a circle) and each node is connected to its *K* nearest neighbors on one side, with this we have an average node degree of 2*K*. Next, each edge is rewired to another random node with a probability *p*, if it is an ER network then $p = 1.0$, and therefore all edges are rewired randomly. For a BA network, we start with *K* nodes and add new nodes connected to *K* existing nodes randomly weighted by their current node degree, and this process generates networks with some nodes with high degrees. A representation of the three networks is displayed in Fig. 1b on the right side of the node degree graph.

### Epidemic process

**Figure 2:**
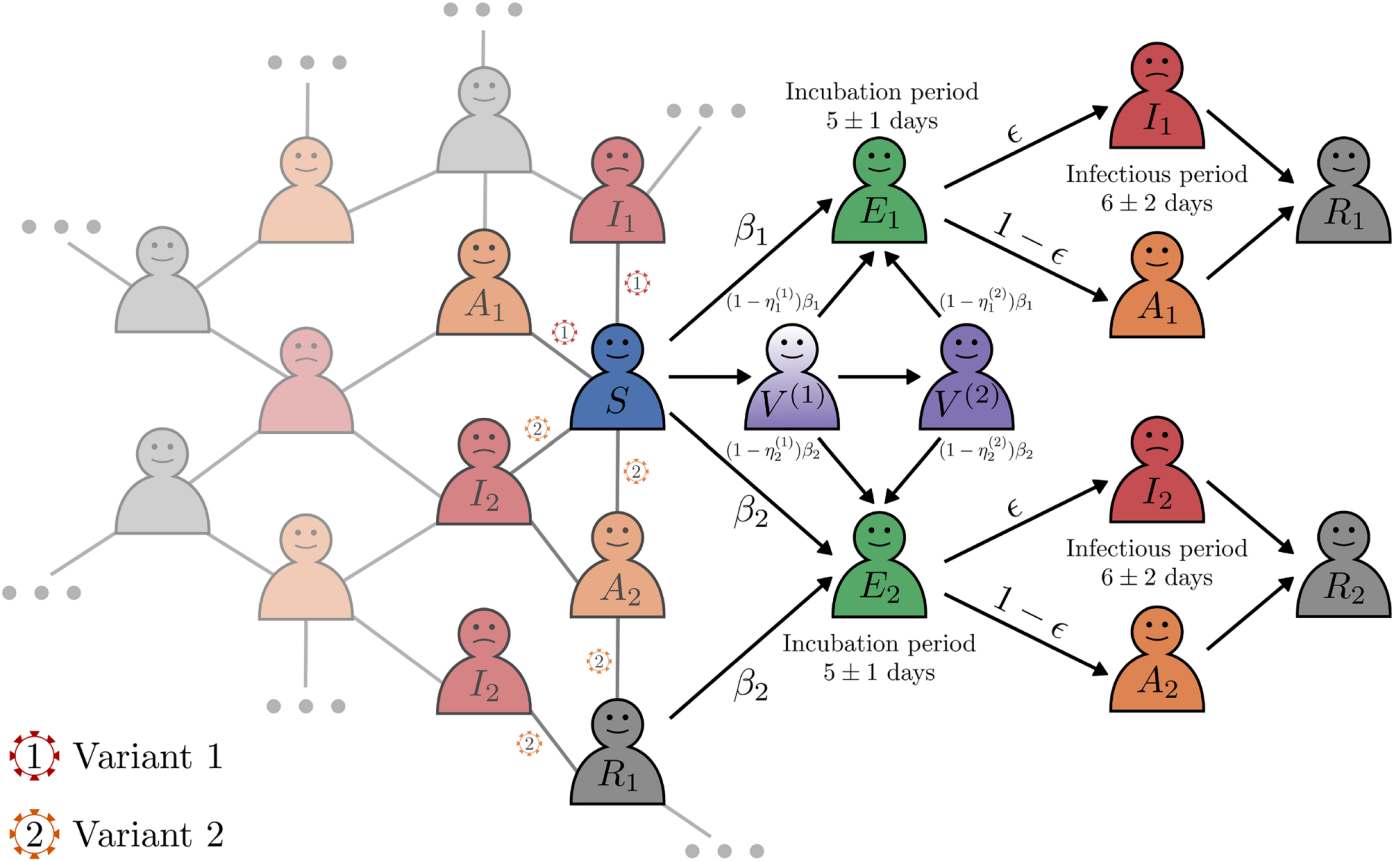
Diagram of the *SEAIRV* model for two virus variants. Black arrows indicate possible state transitions. A susceptible (*S*) node can transition to an exposed ($E_i$) state with a transmission probability $\beta _i$, depending on whether it contacts an infectious node carrying variant 1 ($i = 1$) or variant 2 ($i = 2$). The exposed state lasts for an incubation period of $5 \pm 1$ days. After that, the node may become asymptomatic ($A_i$) with probability $\epsilon$, or infected ($I_i$) with probability $1-\epsilon$, remaining infectious for $6 \pm 2$ days before transitioning to a recovered state ($R_i$). Recovered nodes from variant 1 ($R_1$) can become exposed to variant 2 ($E_2$) with probability $\beta _2$ upon contact with $A_2$ or $I_2$ nodes. In addition, a susceptible node can be vaccinated, receiving a first dose and a second dose 21 days later, entering a vaccinated state ($V^j$), where *j* is the dose number. In this state, the susceptibility to infection of the node is modified to $(1 - \eta _i^j)\beta _i$, reflecting the effectiveness of the vaccine.

In this study, we used a *SEAIRV* model for the epidemic process where the internal state of each node can be Susceptible (*S*), Exposed (*E*), Asymptomatic (*A*), Infected (*I*), Removed (*R*), and Vaccinated (*V*). At the beginning of the simulation, all nodes are *S* except one labeled $I_1$, which is infected by virus variant 1, designated as a wild-type variant. Virus variant 2, a vaccine-resistant mutant virus, is introduced 220 days after the beginning of the simulation by infecting one susceptible node, labeled $I_2$. The *S* nodes in contact with the *A* or *I* nodes can be infected by the virus and enter an $E_1$ or $E_2$ state, depending on the variant transmitted. The $E_1$ or $E_2$ nodes can either develop the disease and go to the $I_1$ or $I_2$ state, respectively, or not develop the disease and go to state $A_1$ or $A_2$, respectively. Eventually the *I* and *A* nodes go to state $R_1$ or $R_2$, depending on the variant transmitted. The $R_1$ nodes can be infected with variant 2 of the virus and go to the $E_2$ state. Vaccinated individuals with one or two vaccine doses are represented by $V^{(1)}$ and $V^{(2)}$, respectively. In our approach, only the susceptible nodes are vaccinated. Figure 2 shows the scheme of the process described above. Following the introduction of the second variant, the simulation continues until there are no more asymptomatic or infected nodes. In other words, the simulation concludes when there are no remaining nodes capable of spreading the infection which marks the endpoint of the process.

The dynamics of an epidemic involves critical considerations related to the transitions in the states of individual nodes within a network. Initially, a susceptible (*S*) node can shift to one of two possible states, $E_1$ or $E_2$. This state transition depends on the contact of the node with an infected or asymptomatic carrier of two different virus variants, denoted variant 1 and variant 2. The mechanism governing this transition is modeled by a Bernoulli process represented by the random variable $B(1, \beta )$, which takes the value 1 to indicate infection of the *S* node and 0 otherwise. Here, $\beta$ is the probability of transmission of the disease, which includes factors such as the rate of interaction between individuals in the population and the inherent contagiousness of the virus. It varies depending on the type of contact: $\beta _1$ is used if the contact is with an $A_1$ or $I_1$ node, and $\beta _2$ if the contact is with an $A_2$ or $I_2$ node. Several studies^39,40^, have conducted analyses to estimate this parameter. In our study, we predetermined the values of $\beta$ for each variant, setting $\beta _1 = 0.022$ for variant 1 and $\beta _2 = 0.025$ for variant 2, using fixed values similar to those from a previous paper^36^. An $R_1$ node can be infected with a $\beta _2$ probability if it is exposed to $A_2$ or $I_2$ nodes thus changing its state to $E_2$. Moving forward, the $A_1$ and $A_2$ nodes represent individuals who were exposed to the virus but did not develop the disease and are considered to be the asymptomatic population. Asymptomatic carriers play a significant role in the transmission dynamics of the virus with studies suggesting that up to 59% of all transmissions may occur from individuals who do not exhibit symptoms^41^. In addition, a proportion of exposed individuals, may eventually develop severe symptoms and contribute to the statistics of confirmed infected cases (*I*). Research estimates this proportion to be around 20%^42,43^, leading us to assign a fixed probability value of $\epsilon = 0.2$ for the development of severe disease in exposed carriers. Consequently, an asymptomatic individual, whether carrying variant 1 ($A_1$) or variant 2 ($A_2$), who does not develop symptoms will be reclassified to the recovered state for their respective variant ($R_1$ or $R_2$). An important aspect of the epidemic model is the length of time that individuals remain in the *E* state and *A* or *I* states before transitioning to the *R* state, which affects the spread and control of the disease. In the literature, it has been reported an incubation period of $5 \pm 1$ days and $6 \pm 2$ days being infectious^41^. Therefore, we configured the nodes with $\Gamma$-distributed waiting times for the *E* states (incubation period distributed as $\Gamma (25,5)$) and for the *A* and *I* states (infectious period distributed as $\Gamma (9,1.5)$). In this model, we assume that asymptomatic individuals have the same infectious period as infected individuals. The *I* and *A* nodes change their states to $R_1$ or $R_2$, respectively, when the waiting time is over, and they are no longer infectious. The time step of the simulations was of 1 day which means that the states of the nodes are updated per day according to the rules above described. The simulation code is stopped when there are no more *A* nodes and *I* nodes.

The dynamics of disease progression involving two variants can be succinctly captured by a set of mathematical expressions that describe the probabilistic transitions between different states of a node within the network. The equations, where $i \in \{1, 2\}$ denotes the variant type, are as follows:

1. Transition from Susceptible to Exposed:
  $$\begin{aligned} P(S \rightarrow E_i) = {\left\{ \begin{array}{ll} \beta _i, &{} \text{if contact with } A_i \text{ or } I_i,\\ 0, &{} \text{otherwise,} \end{array}\right. } \end{aligned}$$
2. Progression from Exposed to Asymptomatic:
  $$\begin{aligned} P(E_i \rightarrow A_i) = {\left\{ \begin{array}{ll} 1-\epsilon , &{} \text{after a period distributed as } \Gamma (25, 5),\\ 0, &{} \text{otherwise,} \end{array}\right. } \end{aligned}$$
3. Progression from Exposed to Infected:
  $$\begin{aligned} P(E_i \rightarrow I_i) = {\left\{ \begin{array}{ll} \epsilon , &{} \text{after a period distributed as } \Gamma (25, 5),\\ 0, &{} \text{otherwise,} \end{array}\right. } \end{aligned}$$
4. Removed from Asymptomatic State:
  $$\begin{aligned} P(A_i \rightarrow R_i) = {\left\{ \begin{array}{ll} 1, &{} \text{after a period distributed as } \Gamma (9, 1.5),\\ 0, &{} \text{otherwise,} \end{array}\right. } \end{aligned}$$
5. Removed from Infected State:
  $$\begin{aligned} P(I_i \rightarrow R_i) = {\left\{ \begin{array}{ll} 1, &{} \text{after a period distributed as } \Gamma (9, 1.5),\\ 0, &{} \text{otherwise,} \end{array}\right. } \end{aligned}$$
6. Cross-variant Reinfection:
  $$\begin{aligned} P(R_1 \rightarrow E_2) = {\left\{ \begin{array}{ll} \beta _2, &{} \text{if contact with } A_2 \text{ or } I_2,\\ 0, &{} \text{otherwise,} \end{array}\right. } \end{aligned}$$

### Lockdown simulation

In the next series of simulations, we implemented two different strategies of lockdown: continuous lockdown and intermittent lockdown. The lockdown is activated if there are 7 days of continuous increase in infected node cases (this condition is met when the newly infected cases reach around 500 per day). We implemented the lockdown strategy differently in different network models: for WS networks, lockdown was simulated by limiting random contacts while preserving local connections. For ER and BA networks, lockdown was simulated by removing a certain percentage of random edges with a 50% reduction in connectivity. The duration of the continuous lockdown scenarios was varied by testing periods of 30, 90, or 150 days. During these periods, a portion of the connections were removed to reflect restricted contacts which were then restored at the end of the lockdown. In the case of intermittent lockdowns, we explored scenarios of 7, 15, or 30 days. This approach involved alternating periods of restricted connectivity and normal interaction of equal duration and repeating this cycle until a total of 180 days was reached marking the end of the lockdown phase.

### Vaccination process

The vaccine is effective against the wild-type strain while the mutant strain evades immunity induced by the vaccine. We assume here that individuals who have recovered from the wild-type infection are still prone to infection with the vaccine-resistant variant of the virus. Regarding SARS-CoV-2, experimental observations highlight that vaccine effectiveness for the wild-type variant within two to four weeks after two doses of vaccine for the prevention of symptomatic COVID-19 is $95\%$ and that for the omicron variant it is $65\%$^44^. However, vaccine efficacy drops in the omicron variant reaching as low as $8.8\%$ at 25 weeks or more after the second dose^44^. In our study, we simulate a vaccination campaign targeting only susceptible nodes within the networks. This campaign is initiated following a trend of continuous increase in new infected cases over a 7-day period. Although it is considered that the vaccine reaches its maximum efficacy two to four weeks after the second dose, before that time, even with one dose, the vaccine can reach an efficacy of up to 65% in two weeks after the first dose, according to work reported in the literature^45,46^. Therefore, in our simulations, we assumed that it would take one week after vaccination for the vaccine to achieve a 65% reduction in the probability of infection. Mathematically, this is represented by adjusting the probability of infection from an infected (*I*) node to a susceptible (*S*) node to $(1-\eta _i^j)\beta _i$, where $\eta _i^{(1)}=0.65$ denotes the vaccine efficacy after the first week after the first dose, $i \in \{1, 2\}$ denotes the virus variant, and $j \in \{1, 2\}$ indicates the dose number. At 21 days after the first dose, if the node remains uninfected, the second dose is administered^46^ increasing the efficacy of the vaccine to its maximum potential. The probability of infection is then further reduced to $(1-\eta _i^{(2)})\beta _i$ where $\eta _1^{(2)}=0.95$ indicates the effectiveness against variant 1 after the second dose. The effectiveness against variant 2, $\eta _2^{(2)}$, was varied as described in the following subsections.

*Variation of the duration of a vaccination campaign* We implemented mass vaccination for six scenarios, vaccinating 10%, 5%, 2.5%, 1.25%, 0.625%, and 0.3125% of the population per day until there were no unvaccinated susceptible nodes. To clarify within our simulations, the same quantity of susceptible nodes is vaccinated daily, and this number corresponds to the various population percentages previously mentioned. This approach was chosen for consistency across all simulations. However, it is important to note that the total number of nodes that ultimately become vaccinated varies among simulations depending on the specific dynamics of the pandemic. The main objective is to evaluate the effectiveness of various vaccination strategies across the three networks. To accomplish this, we focused on the simplest scenario involving just one virus variant.

*Varying vaccine effectiveness* As a secondary objective, we aimed to explore the dynamics of the pandemic when a vaccine-resistant virus variant is introduced in a vaccinated population. However for the virus variant 2 we need to determine the fold change in vaccine efficacy between the wild-type and the mutant strain. We can find a parameter $\kappa$ such that $\kappa = \eta _2^{(2)}/\eta _1^{(2)}$ is the ratio of the vaccine efficacy $\eta _2^{(2)}$ of the variant 2 or mutant strain to the variant 1 or wild-type vaccine efficacy $\eta _1^{(2)}$. To mimic the experimental data in Andrews et al.^44^, we find a $\kappa$ for each week interval presented in Table 1. In this next stage, we hypothesize that the vaccine efficacy for the wild-type ranges from $\lbrace {40\%, 60\%, 80\%,100\%\rbrace }$, and therefore we have the vaccine efficacy for the resistant variant using the $\kappa$-ratio in the Table 1.

*Testing the vaccination approach* Finally, we investigated two different vaccination approaches, a randomized vaccination campaign and a vaccination prioritizing the nodes with the highest degrees, also called hubs. For this objective, we will use the BA scale-free network, as it has been suggested that a targeted immunization of hubs can mitigate the pandemic^37^. To compare these two vaccination strategies we simulated the BA network with an average node degree varied to 8, 12, 16, 20, and 24. At the beginning of each simulation, some nodes are vaccinated with one dose and after 21 days they receive the second dose if they were not infected before. This number of initial vaccinated nodes was varied in 0, 100, 1000, 10,000, and 100,000, being the last one the 10% of the population. In the first vaccination approach, the nodes to be vaccinated are chosen randomly and in the second approach, the nodes are ordered by their degree and then vaccinated in that order. In this case, we focused on the simplest scenario involving just one virus variant.

### Computing

We developed the simulation code in C and took advantage of the high performance computing on the Falcon supercomputer^47^. Falcon is located at the Idaho National Laboratory Collaborative Computing Center (C3) in Idaho Falls, Idaho, USA. It currently consists of approximately 932 nodes with dual Intel Xeon E5-2695v4 18-core processors running at 2.1 GHz (36 cores per node) capable of more than 1 PetaFLOPS of processing power. In total, we ran 100 simulations for each case study using different random number seeds. The duration of each simulation ranged from 1 to 15 minutes depending on the progression of the disease within the network. Some simulations did not result in an epidemic while others extended over a longer period of time.

## Results

### Disease progression in complex networks

**Figure 3:**
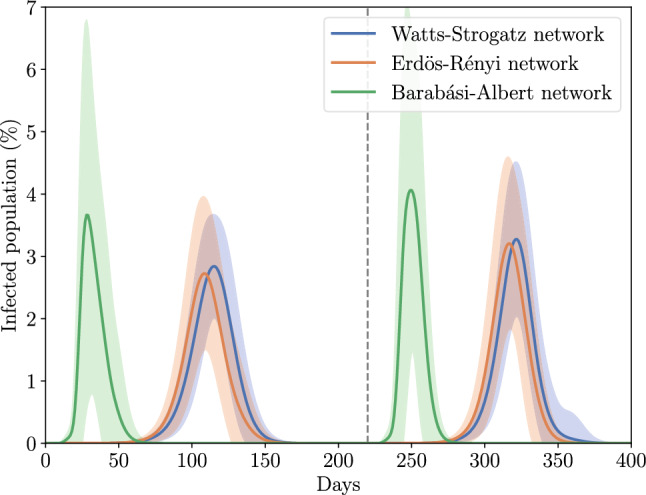
Dynamics of the active infected cases during the course of the epidemic for three different networks. The continuous lines are the average over 100 simulations and the light colors fill double the standard deviation of the data. The grey dashed line indicates the day of the introduction of the variant 2.

In the first series of simulations, we compared the dynamics of the infection for the three types of networks. In Fig. 3, we can observe the percentage of active *I*-nodes (the WS network displayed is with $p = 0.4$) during the course of the epidemic for the three different networks with an average degree of 24. The continuous lines are the average of cases per day and the light colors fill double the standard deviation of the data. The grey dashed line indicates the day of the introduction of the variant 2. We can note that the BA network has a high variability between repetitions and can be seen in its standard deviation which is larger than the other two networks, also its peak is reached earlier than the other networks and is higher. This is because some nodes have a very large degree, thus, in some simulations, these nodes are quickly reached by the infectious nodes (or even the initial *I*-node is one of these) and trigger a rapid transmission of the disease through the network; other simulations do not reach to these nodes and the *I*-nodes end up cleaned out of the network. There is variability among replicates and this is particularly evident in BA networks. Here, we discern well-defined outcomes: a significant proportion closely resembles the primary peak, another portion exhibits a delay and minor peak, and yet another subset experiences a scenario where the pandemic fails to spread. These diverse outcomes result in a broader shaded region with the simulations featuring minor peaks contributing to its shape. On the other hand, WS and ER networks have similar dynamics, especially in infected cases with the 2 variants, therefore disease transmission within these networks is similar if the virus is highly contagious regardless of the fraction of random contacts. In addition, the curve associated with the BA network is observed to be more condensed than those of the other two network types. The disease was eradicated approximately 50 days after the initial increase in cases, as indicated by the narrower span of the curve. In Figure S1 from the supplementary material is displayed a matrix of the average peak of infected cases reached by the variant 1. There are four different WS networks where the difference is in the probability of rewired using $p = 0.2, 0.4, 0.6,$ and 0.8, and the ER and BA networks. The average degree was varied for all networks in 16, 24, 32, and 40. As would be expected, the BA network reaches the highest peak of infected nodes when the average degree is higher except when the average degree is higher where it was slightly lower than the other two networks. The lowest values are when the average degree is low for the WS networks being lower than 2% of the population. We did not see big differences between the WS and ER networks. Table 2 presents the average total number of infections over the entire pandemic for the three networks. Interestingly despite BA networks exhibiting a higher peak compared to the other two networks, they had a lower overall number of infections throughout the entire pandemic. The WS network had the highest total number of infected nodes.

### Lockdown strategies in complex networks

**Figure 4:**
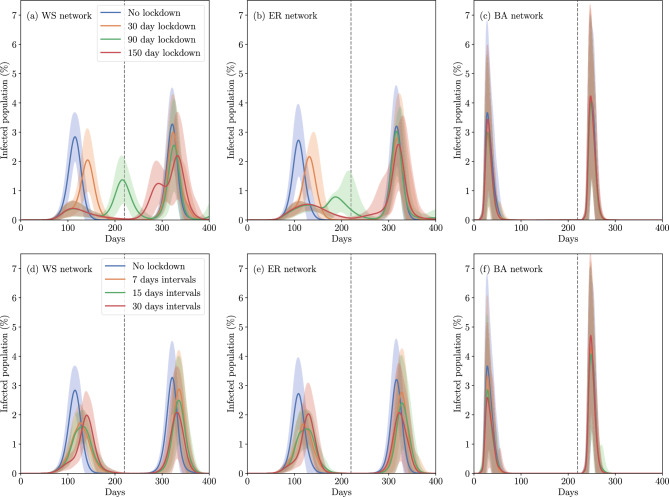
Dynamics of the active infected cases during the course of the epidemic for two different lockdowns: continuous (**a**–**c**) and by intervals (**d**–**f**). The solid lines represent the average across 100 simulations, while the shaded regions indicate two times the standard deviation of the data. Panels (**a**) and (**d**) present the results for WS networks with a parameter of $p = 0.4$, panels (**b**) and (**e**) display the results for ER networks with a constraint involving 50% of the nodes during the lockdown, and panels (**c**) and (**f**) show the outcomes for BA networks with a similar constrain of 50% of the nodes. The grey dashed lines indicate the day of the introduction of the variant 2.

**Scenarios of lockdown in WS networks** In Fig. 4a,d, we presented the results of WS networks with $p = 0.4$. Figure 4a shows the continuous lockdown that lasts 30, 90, and 150 days after the condition is met. The 40% of the edges are retaining (local contacts). We found that lockdowns of 30 or 90 days were not sufficient to eradicate the disease from the population. Instead, the peak of infected cases was delayed in time and reached a lower maximum compared to scenarios without lockdown measures. The introduction of a second viral variant resulted in lower peak infection levels due to competition between the two variants, which effectively reduced the pool of susceptible nodes available to each variant compared to scenarios in which each variant spread independently. Consequently, this inter-variant competition, particularly with the emergence of more transmissible forms alongside existing ones, contributed to a reduction in the daily peak levels of infected cases. In addition, smaller third peaks were observed following the surge attributed to the second variant indicating subsequent, smaller waves of infection. Figure 4d shows the result for an intermittent lockdown with lapses of 7, 15, or 30 days on and the same number of days off. This lockdown is maintained for 180 days. The implementation of an intermittent lockdown strategy led to significant reductions in both peaks with the 15-day strategy resulting in the lowest peak for variant 1. For networks with a high percentage of random contacts, as shown in Figure S2 where $p = 0.8$, it is possible to avoid the peak of the first variant and even decrease up to 80% the second peak mainly due to the second variant by restricting such random contacts at 15-day intervals. Table 2 shows that the total number of infections in WS networks was lowest when the lockdown was for intervals of 30 days, although the intermittent lockdown scenarios had significantly fewer infections compared to no lockdown.

*Scenarios of lockdown in ER networks* In Fig. 4b,e, we presented the results of ER networks with a random restriction of 50% of the edges for continuous and by intervals lockdown, respectively. We can note that the peaks due to variant 1 are lower and displaced compared with the no-lockdown dynamic, but the peaks due to variant 2 are similar for a continuous lockdown strategy. However, for the lockdown by intervals, both peaks are lower compared to the no-lockdown curve and are similar between them regardless of the duration of the interval. The total number of infections was the lowest for a 7-day lockdown, as we can see in Table 2. Figure S3 shows the result considering the 25% of the edges restricted. We can observe that with a 25% of restriction, the dynamic of the infected cases does not change significantly but maintains the behavior of the no-lockdown dynamic regardless of the lockdown strategy.

*Scenarios of lockdown in BA networks* Finally in Fig. 4c,f, we show the results for the BA network with 50% of edge restriction during the lockdown. The results for 25% of edge restriction are displayed in Figure S3. For this network, there is practically no difference between the strategies of lockdown with 25% of edge restriction since all infection peaks are maintained over time. If the contacts are restricted a 50% the peak position is maintained, but the amplitude is slightly reduced. Despite this result, the duration of the lockdown did not matter, since the peaks have approximately the same amplitude regardless of the strategy taken. In these networks, the total number of infections was similar across all lockdown scenarios, as shown in Table 2, with the 30-day lockdown interval having the lowest average number of infected nodes.

### Vaccination strategies in complex networks

**Figure 5:**
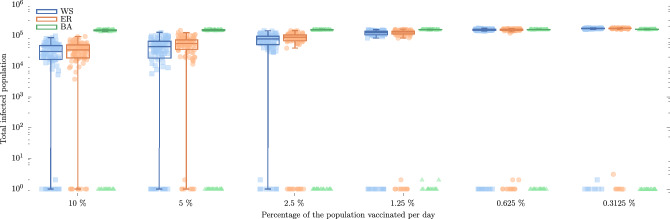
The number of total infected cases with variant 1 considering six different vaccination campaigns. The horizontal axis refers to different vaccination campaigns where the percentage of the population/nodes that were vaccinated per day was varied. Each color represents one of the three network models; all networks were configured with $K=12$ and for the WS network $p=0.4$.

**Importance of time in vaccination campaigns** At this stage, we considered only the infected cases of wild-type virus infection intending to evaluate the efficacy of the different vaccination campaigns. Figure 5 displays the total number of infected nodes until the end of the pandemic for the three types of networks and for six different vaccination campaigns where the nodes were randomly vaccinated. In these plots, each point is the value of the total infected population of one of 100 simulations or replicate for each network and each scenario; points in each case were uniformly distributed over a horizontal range for visualization purposes. The boxplots show the median and the interquartile range of the 100 replicates for each network and each scenario. We do not show the outliers, however these can be inferred from the points. It is noteworthy that in our simulation results, the percentage of the population vaccinated with two doses varied significantly ranging from 98% in the fastest to 30% in the slowest vaccination campaigns for WS and ER networks. This variance is due to nodes that were vaccinated once but became infected before receiving a second dose, and thus were not counted for full vaccination. Despite the higher infection rates in the slowest vaccination scenarios, the percentage of infected cases was approximately 15%, which is still lower than the 17% observed in the no vaccination scenarios (as detailed in Table 2). In contrast, BA networks showed a narrower range of vaccination coverage, with percentages varying from 50 to 20%. This narrower range is due to the rapid spread of the disease in these networks, which quickly reduces the number of susceptible nodes available for vaccination. We can observe a bimodal behavior for all three vaccination campaigns and all three networks and from here we can distinguish between two possible outcomes: a pandemic contraction (less than 100 infected nodes) and a pandemic expansion (more than 100 infected nodes). Considering only the results for WS and ER networks, we observe that in quick mass vaccination campaigns, the cases of infection in the population are lower than those with a slower vaccination campaign, as expected. Now, for the BA networks, we do not observe a very large difference between the different vaccination campaigns. The number of infected nodes reaches the order of $10^5$ nodes. It is worth reiterating that immunized nodes can be infected, even if they already have the two doses. We can also note that some simulations resulted in less than 10 infected cases.

**Figure 6:**
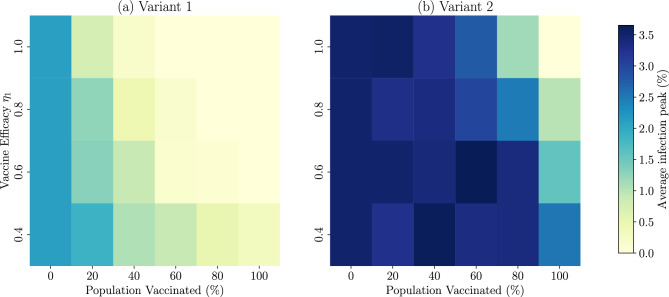
Comparison of average infection peaks at different levels of vaccine efficacy against the wild-type virus ($\eta _1$) within an Erdős–Rényi network. The panel (**a**) shows peaks for variant 1 (wild-type virus); peaks flatten significantly when vaccination coverage exceeds 40% of the population (indicated by the yellow area). The panel (**b**) shows peaks for the more transmissible variant 2; the vertical axis represents vaccine efficacy against wild-type ($\eta _1$), while efficacy against variant 2 ($\eta _2$) is calculated as $\eta _2 = \kappa \eta _1$, where $\kappa$ varies over time as detailed in Table 1. Here the peaks exceed those seen in the no-vaccination scenario, and more than 80% vaccination coverage is required to reduce the peak height of this variant.

*Effect of vaccine efficacy and population covered* Considering the previous results, we decided to use only the ER network. In Fig. 5, we observe that the BA network exhibits less variability in the outcomes across different vaccination campaigns compared to the other two networks. Specifically, the number of infected individuals remains consistently distributed around the same order of magnitude. Conversely, in the WS and ER networks, we observe a broader distribution of results, with the outcomes in the WS network resembling those of the ER network. For this reason, we tested the ER networks here, where the simulation results are more varied compared to the BA networks. For simplification, we consider that a fraction of the population represented in this network was vaccinated at the beginning of the simulations. Figure 6 shows a comparison of average infection peaks at different levels of vaccine efficacy $\eta _1$ against the wild-type virus. Notably, infection peaks for the more transmissible variant 2 (Fig. 6b) are higher than those for variant 1 (Fig. 6a) across all levels of vaccine efficacy. Note that in Fig. 6b the vertical axis represents the vaccine efficacy $\eta _1$ against wild-type, while the efficacy $\eta _2$ against variant 2 is calculated as $\eta _2 = \kappa \eta _1$, where $\kappa$ varies over time as detailed in Table 1. For variant 1, infection peaks flatten significantly when vaccination coverage exceeds 40% of the population, as indicated by the yellow area in Fig. 6a. In contrast, Fig. 6b illustrates that peaks for variant 2 can exceed those of the no-vaccination scenario. This scenario, such as 60% vaccination with $\eta _1 = 0.6$, suggests a larger pool of susceptible nodes available for variant 2, given the higher level of immunity to variant 1. In addition, at least 80% vaccination coverage is required to reduce the peak height of variant 2. Below 40% coverage, there is no significant reduction in the peak of variant 2 infection, regardless of vaccine efficacy. This underscores the need for mass vaccination in response to rapidly evolving and more transmissible viral strains and emphasizes the critical role of widespread immunization regardless of individual vaccine efficacy.

**Figure 7:**
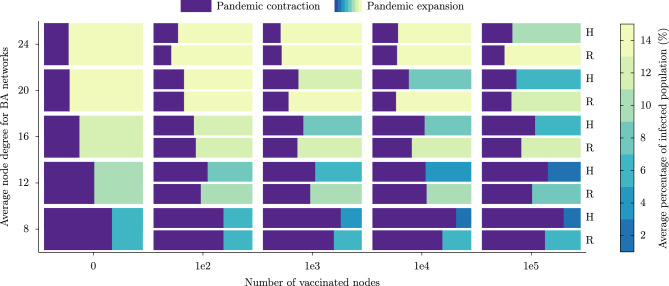
Comparing two immunization strategies to mitigate the pandemic in BA networks. The mosaic plots show results for vaccinated nodes randomly (R), and prioritizing the nodes with the highest degree, also called hubs (H). The vertical axis denotes the average node degree for each simulated network. The horizontal axis denotes the number of vaccinated nodes with the first dose at the beginning of each simulation, they became two-dose vaccinated nodes after 21 days, if they were not infected. We run 100 simulations for each case. The width of each cell represents the total number of simulations conducted, divided into two areas. The purple area on the left indicates the proportion of simulations where the pandemic did not develop (pandemic contraction) leaving fewer than 0.01% of the population infected. The sequential colors area on the right represents the proportion of simulations where the pandemic did spread (pandemic expansion), and the color in the pandemic expansion cells indicates the average percentage of infected population, the average standard deviation of the infected population is around 1.2% (not shown in the plots).

*Importance of vaccinating hubs in scale-free networks* In Fig. 7 we show the results of 100 simulations for each case: a randomized vaccination (R), and a targeting vaccination, prioritizing hubs (H). The width of each cell represents the total number of simulations or replicates for each case, and this width is divided into two areas; the area to the left (purple) represents the proportion of simulations where the pandemic did not develop (a pandemic contraction outcome), and the area to the right (in sequential colors) represent the proportion of simulations where the pandemic did spread (a pandemic expansion outcome). These two outcomes are observed as a bimodal distribution in Fig. 5. From these results, we can observe that the higher the average degree of the network, the lower the pandemic contraction outcome, as expected. Also, we can notice that vaccinating 1% of the nodes does not significantly decrease the total number of infected (sequential colors represent the average percentage of the infected population) but we only notice a slight decrease in the number of infected if we vaccinate 10% of the population. An interesting finding is that the infected population is almost the same whether we vaccinate randomly or by target, differentiating them in the proportion of outcomes. In a scale-free network, if we vaccinate prioritizing hubs then the probability of a pandemic contraction outcome increases.

## Discussion

In this study, we compared three different stochastic networks commonly used to model an epidemic by considering two competing strains of the virus amidst lockdown strategies and vaccination campaigns. Due to the stochasticity of the models, we used a statistic of 100 simulations for each case study where we could observe that some simulations did not develop the epidemic, i.e. we obtained less than 0.01% of the population was infected. By studying the transmission of two variants of the virus in a population with unrestricted contacts, we observed that the dynamic of the epidemic through a population can vary according to the topology of the network. A determining factor is the existence of strongly connected nodes, i.e. nodes with a high degree, as some nodes in a Barabási–Albert network. Figure 3 shows that the BA network had a higher and earlier peak than the other two networks, and this is because nodes with a large number of links are infected and these are the ones that drive the dynamics of the infection among the population. Likewise, the lockdown strategies in BA networks considered here were not enough to mitigate the epidemic and did not allow time for the vaccines to reach their maximum efficacy to prevent further infections. On the other hand, although the BA network had the highest peak of the three networks tested, it consistently had the lowest total number of infected individuals throughout the pandemic in all scenarios. This network structure facilitated the rapid spread of the pathogen, resulting in an early peak of infections. Infections occurred swiftly enough to encompass almost all possible nodes with the disease within a similar timeframe, and the pandemic concluded relatively early due to a high rate of recoveries. Populations that have a structure of strongly connected nodes, such as cities characterized by densely populated local areas that concentrate social interactions in certain locations, have a big challenge in public health measures which must be rigorously applied. On the other hand, in Fig. 4 for BA network type, there were virtually no differences observed between continuous lockdown and interval lockdown strategies. Interestingly, the intermittent lockdown resulted in fewer infected cases in the other two network types (ER and WS), suggesting that intermittent lockdowns could serve as a viable alternative to mitigate the adverse effects associated with prolonged, strict containment measures^48^.

Considering the other two networks, we found interesting results: first, the dynamic of the infected cases is slow and the height of their peaks did not exceed those of the BA networks. Second, when the average degree of the network is increased, the random contacts do not appear to play an important role during the transmission of the disease. The latter suggests that in strongly connected populations, that is, with a large number of contacts per node, clustering is the determining factor controlling pandemic dynamics. In analyzing lockdown strategies, it is clear that the use of a continuous lockdown approach, without achieving complete elimination of the virus from the population during the lockdown period, merely shifts the peak of infection. This suggests that while lockdown may temporarily reduce transmission rates, it does not fundamentally alter the trajectory of the outbreak unless the virus is completely eliminated from the population within the lockdown period. Implementing interval-based lockdowns proved effective in reducing the total number of infected nodes, with the 7-day interval strategy having the lowest number of variant 1 infections, although the 15-day interval lockdown had the lowest overall infection peak. An interesting result is that 150 days of continuous lockdown could not suppress the first peak due to the first variant. It should be clarified that during lockdown we reduce contacts by up to half, so it is expected that a stricter (and ideal) lockdown will eliminate the number of infected cases.

Although some vaccines require a single dose regimen to achieve acclaimed efficacy levels^18^, the majority of vaccines in use require more than one dose to achieve the levels of efficacy claimed by manufacturers^18,49^. In our model, we considered an efficacy of 65% to represent a hypothetical first dose of the Pfizer vaccine where about 90% of the nodes are vaccinated with one dose during the campaign. We found a considerable reduction in the cases of infection, therefore it could be considered that a good vaccination strategy is to prioritize the coverage of the first dose before applying the second dose, especially in countries with emerging economies, as suggested by Kaura et al.^45^. Furthermore, our analysis of Fig. 5 reveals a bimodal distribution within these vaccination campaigns that leads to two distinct outcomes: pandemic expansion and pandemic contraction. Remarkably, simulations with identical parameters yield these divergent results solely due to the inherent randomness in the infection of nodes. In the case of pandemic contraction, fewer than 10 individuals become infected, while in WS and ER networks, pandemic expansion results in approximately $10^4$ or more infections. Interestingly, within the BA network, regardless of the vaccination campaign, the total number of infected individuals in the pandemic expansion outcome exceeds $10^5$ infected cases, as we can see in Fig. 5. This observation hints at the potential presence of influential nodes that play a pivotal role in governing virus transmission throughout the population. Identifying and targeting these nodes could offer a means of controlling infection dynamics during a pandemic. Such nodes might represent individuals with extensive connections, possibly within settings like public transport terminals. Consequently, emphasizing adherence to preventive measures in these locations becomes of paramount importance.

From our numerical simulations, we have found that when a vaccine-resistant strain is in circulation with the wild-type of the virus in the population, vaccination plays a major role in achieving eradication. The strategies of vaccination tested in Fig. 6 showed some obvious results, and as we expected vaccinating as many people as possible in the shortest time is the best way to avoid further contagion, although, of course, this would be an unrealistic vaccination campaign. In addition, more people are infected with the resistant strain than the wild-type irrespective of the efficacy of the vaccine and the population coverage. An efficacy of 0.8 or higher yields low to no infections with the wild-type strain when 40% or more of the population is vaccinated. This observation is due to the increased transmissibility and reduced efficacy of the vaccine towards the mutant strain^44,50^.

Scale-free networks are characterized by having highly connected nodes, and it has been suggested strategies of lockdown and immunization of targeting the hubs^14,37^. However, these studies consider networks with less than one million nodes, the average degree of nodes less than 10, or the connections of the hub nodes are partially or totally eliminated when a mitigation strategy is simulated. In real networks, the connections between nodes are not completely eliminated, i.e. an immunized node can be infected and in turn infect other nodes although with less probability. Our results presented in Fig. 7 show that in these types of scale-free networks, we cannot reduce the number of infected using a vaccination strategy focused on hub nodes, but we can increase the probability that the pandemic does not develop. If we vaccinate at least 0.1% of the nodes, prioritizing their degree of connectivity, we could achieve a probability of more than 50% for a pandemic contraction scenario if the average degree of the network is less than 16.

## Conclusions

Our study presents a comprehensive analysis of epidemic dynamics across three distinct stochastic network models that incorporates factors like competing viral strains, lockdown strategies, and vaccination campaigns. The findings underscore the complex interplay between network topology and epidemic outcomes; also they highlight the key role of highly connected nodes in the spread of infection, particularly within Barabási–Albert networks. These networks demonstrated an early and higher peak in infections but ultimately exhibited the lowest overall infection count, suggesting rapid transmission dynamics. Intermittent lockdown strategies, especially with 7-day intervals, showed promise in reducing the total number of infection cases, offering a feasible alternative to a longer continuous lockdown. When simulating vaccination campaigns, we observed a bimodal distribution leading to two distinct outcomes: pandemic contraction and pandemic expansion. For BA networks, differences between vaccination strategies were minimal. We modeled vaccination scenarios that took into account viral evolution toward a more transmissible virus strain, adjusted for declining efficacy against a new variant, and found that while over 40% coverage flattened wild-type virus peaks, at least 80% was needed to reduce peaks for variant 2, highlighting the critical need for mass vaccination regardless of individual vaccine efficacy. Our results suggest that in scale-free networks, vaccination strategies focused on hub nodes may not significantly reduce the number of infections but may improve the chances of averting a pandemic outbreak.

### Supplementary Information

Supplementary Information.

## Data Availability

The datasets generated and/or analyzed during the current study are available in the GitHub repository, https://github.com/systemsmedicine/covid-network.
