# Supplementary material for: Assessing the impacts of vaccination and viral evolution in contact networks
Source: Sci Rep. 2024 Jul 8;14:15753. doi: 10.1038/s41598-024-66070-5 (PMC11231155; doi:10.1038/s41598-024-66070-5)
Supplement: Supplementary file 1 — Supplementary Information. [file 41598_2024_66070_MOESM1_ESM.pdf]

Assessing the impacts of vaccination and viral evolution in contact networks

Rodolfo Blanco-Rodríguez[1,2], **Josephine N.A. Tetteh**[3], **and Esteban Hernández-Vargas**[1,2,*]

[1]Department of Mathematics and Statistical Science, University of Idaho, Moscow, 83844–1103, Idaho, USA
[2]Institute for Modeling Collaboration and Innovation, University of Idaho, Moscow, 83844–1103, Idaho, USA
[3]Frankfurt Institute for Advanced Studies, Frankfurt am Main, 60438, Germany
[*]esteban@uidaho.edu

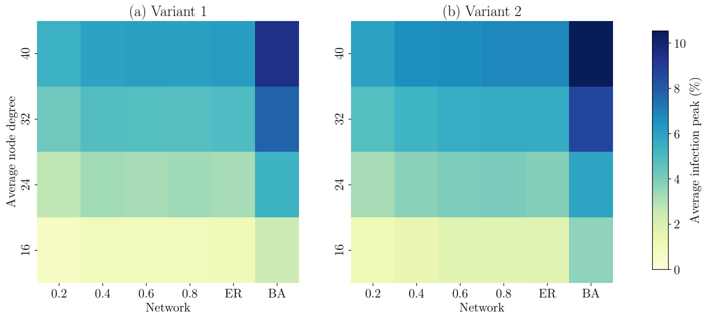

Figure S1. Matrix of the average peak of infected cases reached by (a) variant 1 and (b) variant 2 for the three different network models as horizontal axis and different average degrees as vertical axes. The fraction p of random contacts in Watts-Strogatz (WS) networks was varied to 0.2, 0.4, 0.6, and 0.8. The Erdös-Rényi (ER) network corresponds to $p = 1.0$, i.e. a completely random network. The results for the Barabási-Albert (BA) network are added next to the ER network. The results are an average of 100 simulations.

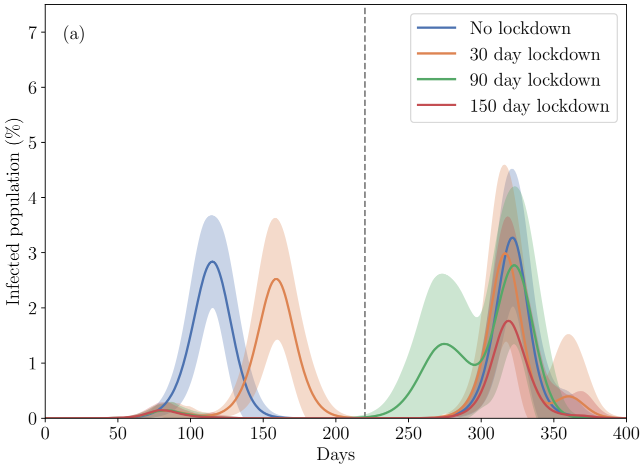

Figure S2. Dynamics of the active infected cases during the course of the epidemic for WS networks and two different lockdowns: continuous (panel a) and by intervals (panel b). The continuous lines are the average over 100 simulations and the light colors fill double standard deviation of the data. p is the probability of nodes to be rewired.

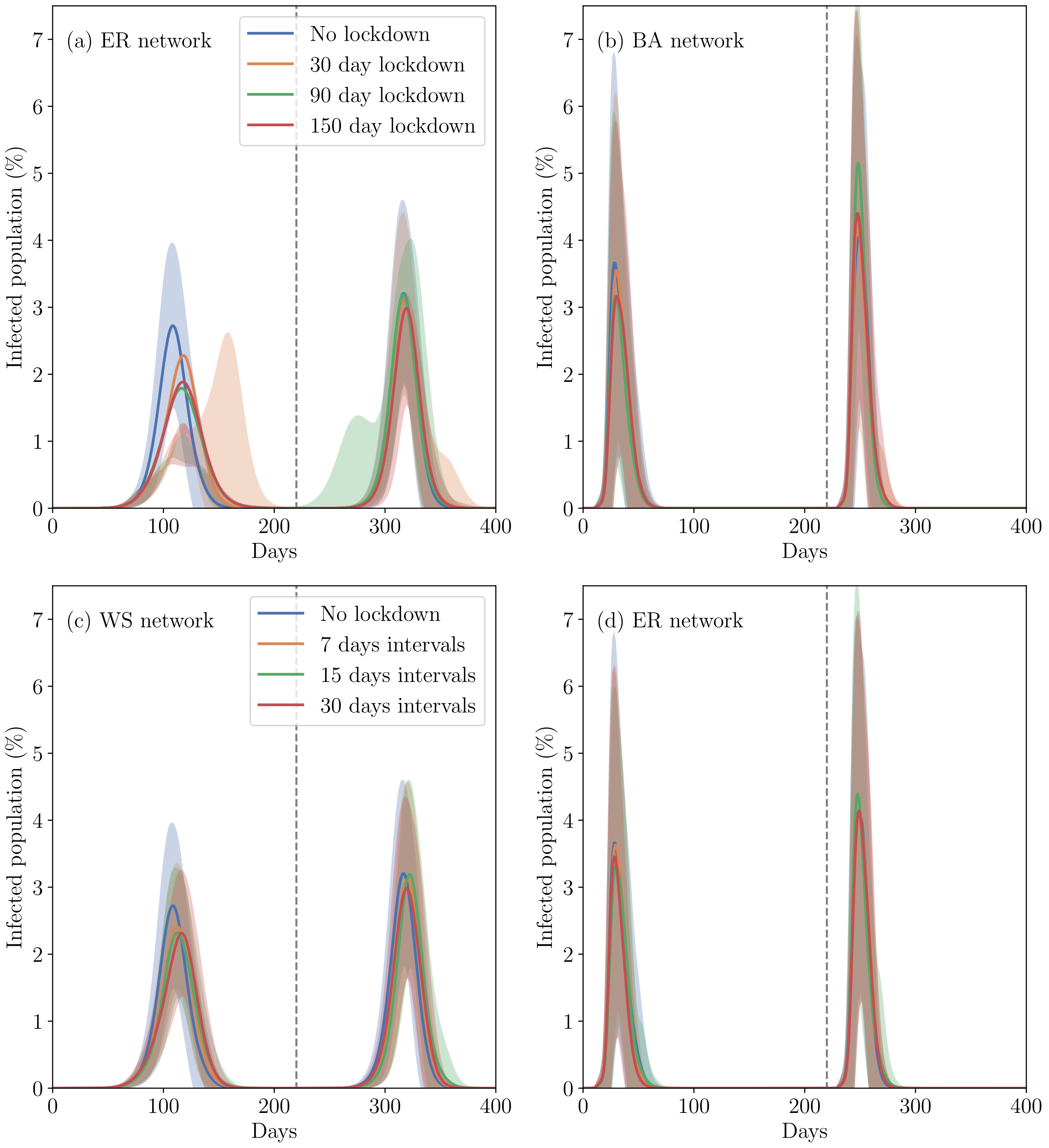

Figure S3. Dynamics of the active infected cases during the course of the epidemic for ER and BA networks and two different lockdowns: continuous (panels a and b) and by intervals (panels c and d). The continuous lines are the average over 100 simulations and the light colors fill double the standard deviation of the data. These plots show results for a restriction of 25% of the nodes during lockdown.
